# Mitral Subvalvular Apparatus Intervention In Patients With Obstructive Hypertrophic Cardiomyopathy: does it need?

**DOI:** 10.1186/1749-8090-10-S1-A138

**Published:** 2015-12-16

**Authors:** A Bogachev-Prokophiev, S Zheleznev, M Fomenko, A Pivkin, A Afanasiev, R Sharifulin, A Karaskov

**Affiliations:** 1 Heart Valves Surgery Department, State Research Institute of Circulation Pathology, Novosibirsk, Russian Federation

## Background/Introduction

Systolic anterior motion (SAM) and mitral regurgitation (MR) in patients with hypertrophic obstructive cardiomyopathy (HOCM) is associated with: Venturi effect and small LV cavity. According 2011 ACCF/AHA guideline extended myoectomy is gold standard in treatment patients with HOCM, however mitral subvalvular apparatus (MSA) intervention is still unclear.

## Aims/Objectives

The purpose of this randomize study was assessment MSA intervention during extended septal myectomy in patient with HOCM and moderate to severe MR.

## Method

Between 2010 and 2014, 182 patients underwent of extended myectomy procedures. 70 patient met inclusion criteria: were randomly assigned to receive MSA intervention in addition to septal myectomy (MSI group; n = 36) or undergo septal myectomy only (without MSI group; n = 34). A primary HOCM was the main indication for surgery according to 2011 ACCF/AHA guidelines. Mean age was 52.8 ± 14.2 years (range 22 to 74 years). Mean peak gradient was 90.7 ± 24.2 mm Hg. Mean thickness of interventricular septum was 26.1 ± 4.3 mm. SAM syndrome observed in all patients. MR: moderate - 42 (60%) pts, severe 28 (40%) pts.

## Results

There were no early death. MSA intervention include: mobilization papillary muscle 36 (100%) pts, secondary chords resected 36 (100%) pts (from 2 to 6), longitudinal resection papillary muscles (papillary muscles more than 15 mm) 32 (88.9%) pts and excision of abnormal papillary muscles 9 (25.0%) pts. Residual MR ≤2+ was 14.7% (5 pts) in group without MSA and nobody had in MSA group (p = 0.023); residual SAM was 23.5% (8 pts) in group without MSA and only 1 (2.7%) pts had in MSA group (p = 0.01). Mean time cross clamping was 42.4 ± 15.2 min (without MSA) and 56.4 ± 20.8 min (MSA group) (p = 0.002). Peak LVOT gradient was 12.2 ± 6.3 mm Hg (without MSA) and 8.7 ± 4.5 mm Hg (MSA group) (p = 0.009).

## Discussion/Conclusion

MSA intervention during septal myectomy in patients with HOMC and MR safe and effective procedure. Complex MSA intervention as in addition to septal myectomy allows more effective eliminate SAM-syndrome and MR.

